# A novel sorting method uncovers metabolic heterogeneity between mononucleated and binucleated tetraploid hepatocytes

**DOI:** 10.1016/j.jbc.2026.113324

**Published:** 2026-07-13

**Authors:** Yusuke V. Watanabe, Jonathan H. Sussman, Tomoya Anami, Gaku Shimodaira, Moena Hattori, Saaya Yamane, Masaki Nishikawa, Yasuyuki Sakai, Takeshi Katsuda

**Affiliations:** 1 Department of Bioengineering, Graduate School of Engineering, The University of Tokyo, Tokyo, Japan; 2 Medical Scientist Training Program, Perelman School of Medicine, University of Pennsylvania, Philadelphia, Pennsylvania, USA; 3 Department of Chemical System Engineering, Graduate School of Engineering, The University of Tokyo, Tokyo, Japan

**Keywords:** hepatocyte, ploidy, nucleus, flow cytometry, liver metabolism, liver zonation

## Abstract

Hepatocytes display notable ploidy diversity, varying both in the number of genomic copies and in the number of nuclei. In adult mice, more than 50% of hepatocytes are tetraploid (4n), which can exist as either mononucleated (1x4n) or binucleated (2x2n) cells. Despite this distinction, these two cell types have traditionally been grouped and studied as a single population. One likely reason for this is that conventional ploidy-sorting methods classify hepatocytes based solely on total DNA content, without distinguishing between mononucleated and binucleated states. Consequently, it remains unclear whether 1x4n and 2x2n hepatocytes are functionally equivalent. In this study, we developed a novel FACS strategy to distinguish and isolate 1x4n and 2x2n hepatocytes. Our approach leverages Hoechst-area to assess total ploidy and Hoechst-height to differentiate mononucleated and binucleated hepatocytes. Transcriptome analysis comparing these two populations revealed enrichment of various metabolism-related gene signatures in 1x4n hepatocytes. Importantly, these metabolic gene expression programs were independent of liver zonation, a well-known driver of metabolic heterogeneity in hepatocytes. Our findings uncover a previously underappreciated layer of diversity in the liver and may provide a new framework for studying the physiological and pathological roles of nuclear configuration in polyploid cells in general.

Hepatocytes, which comprise approximately 70 to 80% of the liver cell population, exhibit a unique ploidy status (1, 2). While most other mammalian cell types possess two copies of the genome, a state known as diploidy (2n), hepatocytes often contain more than two copies. In the adult mouse liver, tetraploid (4n) hepatocytes are the most abundant population, followed by octoploid (8n) cells, whereas diploid (2n) cells account for only approximately 10% of the total (3, 4). Whether ploidy differences account for the phenotypic heterogeneity of hepatocytes remains under debate.

Earlier studies have explored the differences between diploid and polyploid hepatocytes, leading to the discovery that polyploidy is involved in hepatocellular carcinoma and aging (5, 6, 7, 8). Because polyploid hepatocytes carry extra copies of the genome, they are more resistant to tumorigenesis caused by loss of heterozygosity (LOH) (5). In addition, polyploid hepatocytes exhibit reduced cell-to-cell transcriptional variability compared with diploid hepatocytes, although increased transcriptional variability is a hallmark of aging (6). In contrast, the expression levels of aging markers, including p53 and p16, are higher in polyploid than diploid cells (7). Other studies suggest that diploid and polyploid hepatocytes are metabolically comparable (5, 8). Collectively, these findings suggest that polyploid hepatocytes contribute to the maintenance of liver homeostasis by preventing alterations in liver structure and function.

In addition to the diversity of cellular ploidy states, polyploid hepatocytes are further classified based on the number of nuclei (9, 10, 11). Specifically, tetraploid hepatocytes exist either as mononucleated cells containing four genomic copies within a single nucleus (1x4n) or as binucleated cells containing two genomic copies in each of two nuclei (2x2n). Similarly, 8n hepatocytes can be classified into 1x8n and 2x4n hepatocytes.

While differences between 2n and polyploid hepatocytes have already been studied, little is known about differences between 1x4n and 2x2n hepatocytes. A major limitation in studying these populations has been the lack of a method to isolate them. In ploidy research, hepatocytes are commonly stained with a DNA-binding dye and distinguished by cellular ploidy (2n, 4n, and 8n) using flow cytometry (9, 12, 13). In this method, both 1x4n and 2x2n hepatocytes are classified as 4n, preventing their comparative analysis. To overcome this limitation, recent advances in transcriptomic technologies have enabled comparisons between 1x4n and 2x2n hepatocytes without sorting live cells, revealing potential functional differences. A single-nucleus RNA sequencing (snRNA-seq) study suggested that the expression profiles of nuclei from 2x2n cells are more similar to those of 1x2n cells than to those of 1x4n cells (14). In another study, single-cell RNA sequencing (scRNA-seq) integrated with microscopy enabled simultaneous measurement of both transcriptome and ploidy states, suggesting that 2x2n hepatocytes are more metabolically active than 1x4n hepatocytes (15). Although these studies using emerging technologies have provided new insights, they also have limitations, including a small number of analyzed cells and low RNA capture efficiency.

In this study, we aimed to develop a method to physically separate 1x4n and 2x2n hepatocytes, which have not previously been isolated independently. We hypothesized that the principle of doublet discrimination could be applied to polyploid hepatocytes to enable their separation. Using this approach, we established a method to isolate these populations and investigated their transcriptional differences by RNA sequencing (RNA-seq).

## Results

### Two-dimensional FACS analysis of nuclear signals resolves 1x4n and 2x2n hepatocytes

**Figure 1:**
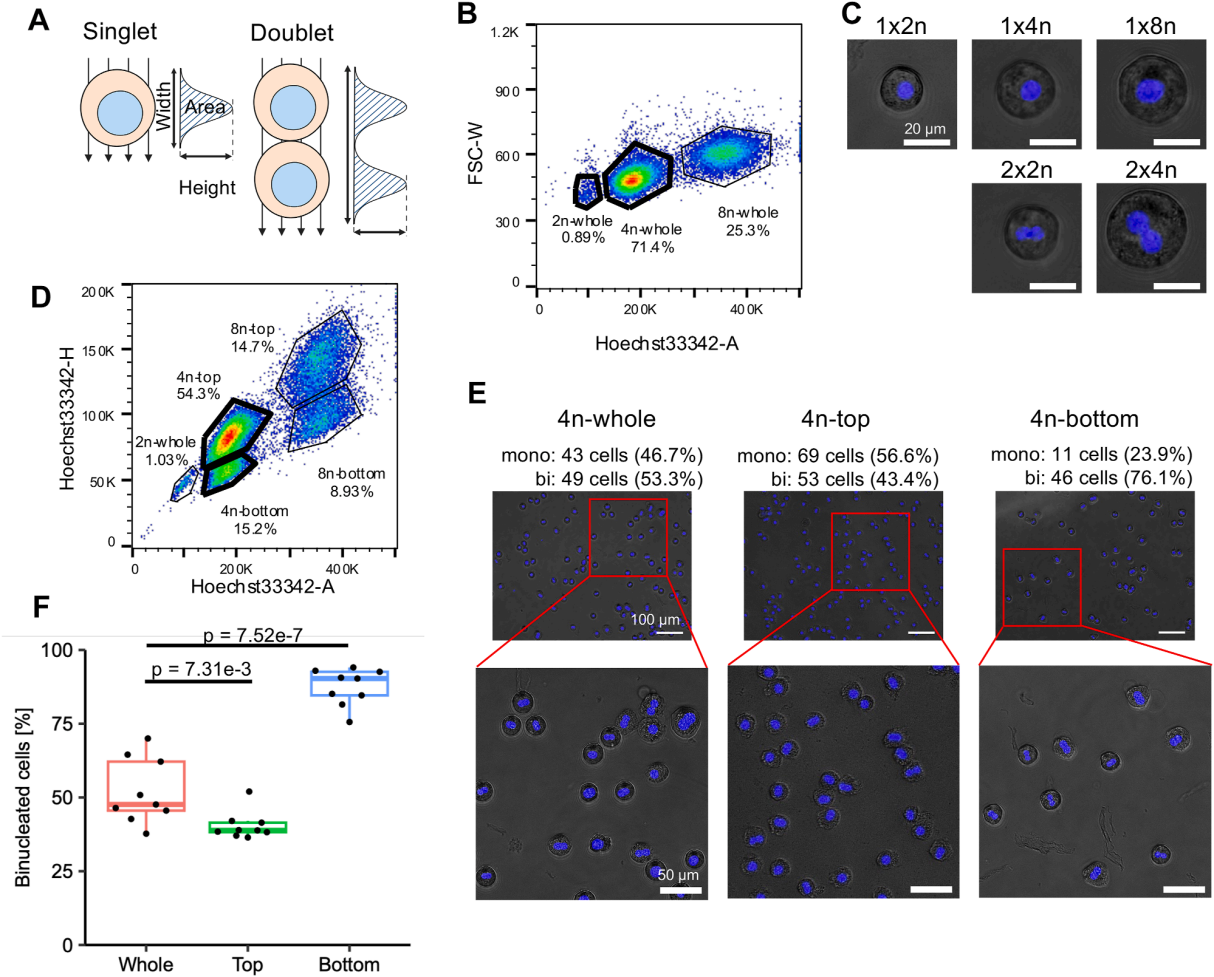
**Development of a novel strategy for isolating 1x4n and 2x2n hepatocytes.***A*, the principle of doublet discrimination in flow cytometry. Width represents signal duration, height represents the peak signal intensity, and area is the integral of width and height. *B*, the conventional gating strategy for sorting 2n, 4n and 8n hepatocytes following the cellular ploidy states. *C*, representative images of sorted hepatocytes with distinct ploidy states. *D*, the newly developed gating strategy for sorting mononucleated and binucleated hepatocytes. 4n hepatocytes are separated into the 4n-top and 4n-bottom populations. The same classification applies to 8n cells. *E*, representative images of plated 4n hepatocytes sorted with the conventional *B* or new *D* gating strategy. *F*, proportions of binucleated cells in each of the sorted 4n populations (n = 9). Significance was assessed using a one-sided Welch’s *t* test. *A* was created in https://BioRender.com.

To fractionate 1x4n and 2x2n hepatocytes using flow cytometry, we hypothesized that the principle of doublet discrimination could be applied. In flow cytometry, three parameters are generally used to detect cell events: the signal duration (width), peak intensity (height), and their integrated value (area) (Fig. 1*A*) (16, 17). Among these, the area parameter is most commonly used for quantification of a given signal due to its stability. In contrast, the width or area together with height parameters of forward scatter are used to identify doublet events. Specifically, the forward scatter-width (FSC-W) and area (FSC-A) values of doublets are twice as high as those of singlet events, while the forward scatter-height (FSC-H) values are similar between doublets and singlets. Therefore, doublets can be filtered by plotting events using FSC-H and FSC-A on two axes and gating events with lower FSC-H values as doublets, excluding them from downstream analyses. Based on this principle, in cell cycle assessment, doublets composed of two G0/G1-phase cells were distinguished from singlet cells in the G2/M-phase (18). Also, in a previous plant study, singlet nuclei of unreduced pollen (1x2n) and doublet nuclei of reduced pollen (2x1n) were stained with the DNA-binding dye propidium iodide and successfully fractionated (19). Consequently, we hypothesized that mononucleated and binucleated hepatocytes were distinguishable by the fluorescence signal from the DNA-binding dye Hoechst 33,342.

First, we confirmed that this conventional gating strategy enabled sorting of mouse hepatocytes with different cellular ploidy states (2n, 4n and 8n) (9, 12, 13). Freshly isolated hepatocytes were stained with Hoechst 33,342 and dead cells with TO-PRO-3. After removing smaller non-parenchymal cells, doublet cells, and dead cells (Fig. S1*A*), live hepatocytes were plotted according to FSC-W and Hoechst-area as the axes (Fig. 1*B*). Consistent with previous studies, hepatocytes were successfully sorted based on cellular ploidy (Fig. S1*B*), yielding mononucleated or binucleated cells whose nuclear sizes increased according to ploidy states (Fig. 1*C*). Notably, both 1x4n and 2x2n hepatocytes, as well as 1x8n and 2x4n hepatocytes, exhibit the same area value because the nuclear volume scales proportionally with DNA content (20).

We next tested the hypothesis that hepatocytes could be separated into mononucleated and binucleated populations. By gating live hepatocytes with Hoechst-height and Hoechst-area, 4n and 8n hepatocytes were separated into two distinct populations, respectively (Fig. 1*D*). Specifically, we classified 4n hepatocytes into 4n-top (higher Hoechst-height) and 4n-bottom (lower Hoechst-height) populations. To examine whether these populations correspond to 1x4n and 2x2n hepatocytes, we sorted the whole 4n (4n-whole), 4n-top and 4n-bottom hepatocytes. The proportions of mononucleated and binucleated cells were determined by microscopy after plating, confirming that 4n-top and 4n-bottom populations were enriched for mononucleated and binucleated cells, respectively (Figs. 1, *E* and *F*). Specifically, the 4n-whole population consisted of 48.0% mononucleated and 52.0 ± 3.7% binucleated cells (mean ± SEM). In contrast, the mononucleated fraction in the 4n-top population increased to 59.6 ± 1.6%, while the binucleated fraction in the 4n-bottom population reached 87.5 ± 2.1% (n = 9). These results indicated that 1x4n and 2x2n cells are enriched in the 4n-top and 4n-bottom, respectively.

### *In silico* analysis models the distribution of binucleated hepatocytes along the Hoechst-height parameter

**Figure 2:**
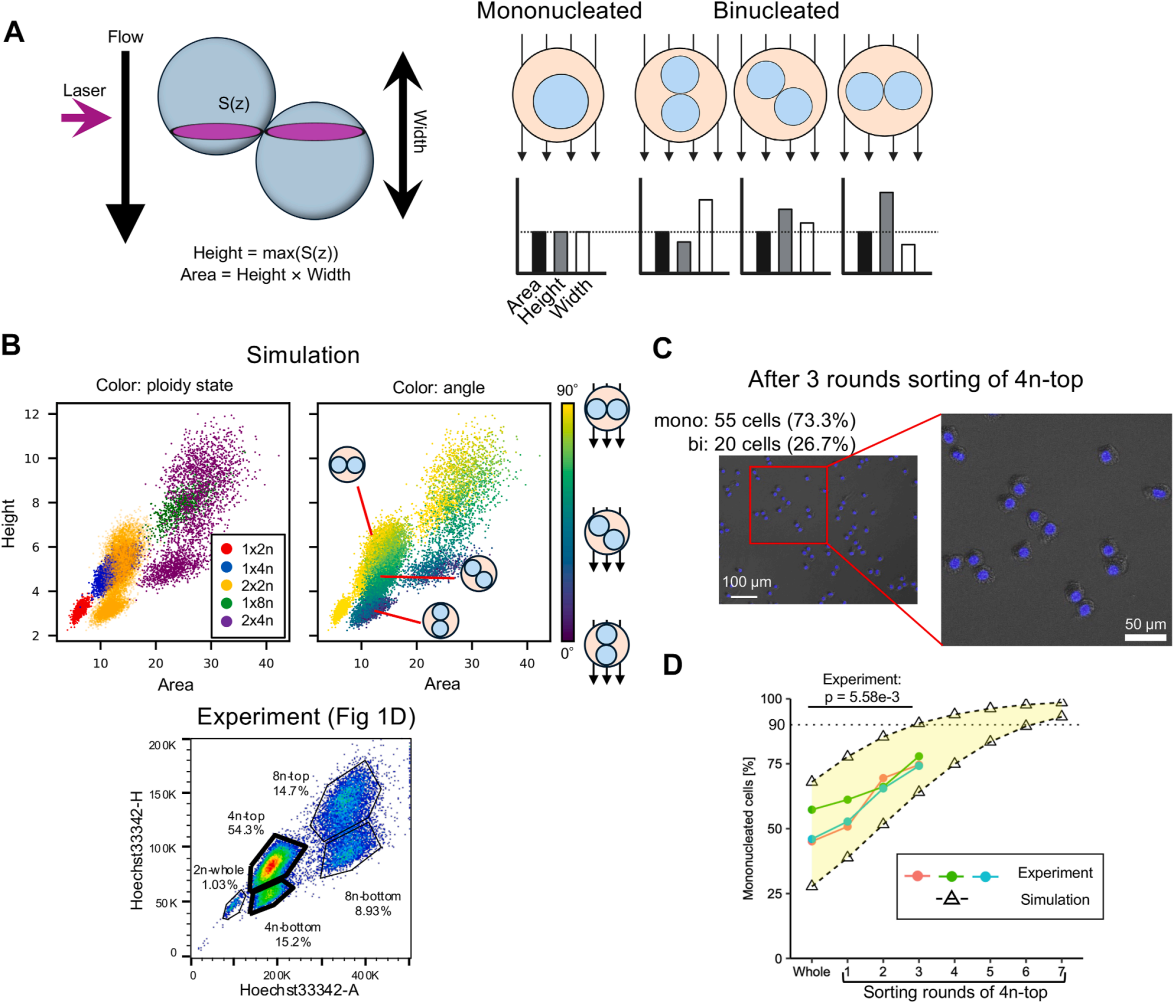
**Examination of the behavior of binucleated hepatocytes in flow cytometry.***A*, (*Left*) a schematic of the model used to examine the hypothesis that nuclei of binucleated hepatocytes can be randomly distributed in flow cytometry. Fluorescence of hepatocytes, with Hoechst 33,342, is represented as two spheres. The width and height values are calculated as signal duration and signal peak, respectively. The area value is the product of width and height. (*Right*) A schematic depicting how area, height, and width change with the orientation of the nuclei. *B*, results of the model. The colors in the upper *left* figure represent ploidy levels. The colors in the upper *right* figure represent the angle between binucleated hepatocytes and flow cytometry. The lower figure is the result of the actual experiment. *C*, a representative image of plated cells after three rounds of sorting of 4n-top hepatocytes. *D*, proportions of mononucleated cells after multiple rounds of sorting of 4n-top hepatocytes (n = 3). The experimental results are shown as solid lines while the simulation results are shown as *dashed lines*. Points are the actual results of the experiment or simulation. The area between triangles indicates the 95% confidence interval and shaded in yellow. Significance was assessed using a one-sided Welch's *t* test. (*A*) was created in https://BioRender.com.

Next, we sought to understand why only ∼60% of 1x4n hepatocytes were enriched in the 4n-top population, whereas 2x2n hepatocytes were enriched to nearly 90% in the 4n-bottom population. We hypothesized that binucleated cells might appear in both the 4n-top and 4n-bottom populations, depending on the orientation of the two nuclei (Fig. 2*A*). Specifically, if the nuclei of the binucleated cells were aligned with the flow direction, the height value would be detected lower, whereas if the nuclei were orthogonal to the flow direction, the height value would be detected higher. It should be noted that mononucleated hepatocytes would exhibit specific values under this hypothesis.

To test this hypothesis, we created an *in silico* model in which the angle between two nuclei of 2x2n hepatocytes and flow was randomly assigned with a uniform distribution (Fig. 2*A*). The nuclear volume was assumed to be proportional to both cellular ploidy and nuclear ploidy: *e.g.*, the nucleus of a 1x4n cell had twice the volume of the nucleus in a 1x2n hepatocyte, while each nucleus of 2x2n cells had the same volume as the nucleus in a 1x2n cell. Therefore, the total nuclear volumes of 1x4n and 2x2n cells were the same. The width value was defined as the length of nuclei along the direction of flow. The maximum area among the planes orthogonal to the flow direction was taken as the height value. Then, the area value was approximated as the product of height and width. The proportion of each ploidy level was based on the experimental result (Fig. 1*B*, left in Fig. 1*E* and left in Fig. 1*F*). As a result, the scatter plot generated by this model was very similar to the actual experimental result (Fig. 2*B*). Notably, when two nuclei were parallel with flow, the height values were lower than those of mononucleated hepatocytes, but when two nuclei were orthogonal with flow, the height values were as high as those of mononucleated hepatocytes. This model supported our hypothesis that the height value of binucleated cells did not always remain smaller than that of mononucleated cells but instead varied depending on the angle.

If the orientation of two nuclei is probabilistic rather than an inherent property of each cell, 1x4n hepatocytes would be enriched in the 4n-top population by repeating sorting of this population with flow cytometry because 2x2n hepatocytes would be removed from the 4n-top population probabilistically. Indeed, experiments demonstrated that three rounds of sequential sorting gradually increased the proportion of 1x4n cells to 75.5 ± 1.2% (n = 3) (Figs. 2, *C* and *D*). Finally, we examined whether the *in silico* model could recapitulate the experimental results (Fig. 2*D*, Fig. S2). The population of mononucleated cells in the 4n-whole fraction (Fig. 1*F*) was fitted to a normal distribution, and the behavior of events within the 95% interval was examined. The simulation also demonstrated a gradual increase in the proportion of mononucleated cells, reaching over 90% after three to seven rounds of enrichment.

Collectively, these results provided a mechanistic explanation for the distribution of binucleated hepatocytes along the Hoechst-height axis and validated our gating strategy for selective enrichment of 1x4n and 2x2n hepatocytes.

### Cellular ploidy rather than nuclear configuration drives transcriptional differences in hepatocytes

Using this sorting method, we next investigated differences in gene expression profiles among hepatocytes with different ploidy states. We sorted 2n-whole, 4n-whole, 4n-top and 4n-bottom populations and performed RNA-seq (metadata in Table S1). For 4n-top hepatocytes, we used cells after a single round of sorting, rather than repeated sorting, to ensure enough cells for RNA-seq.

**Figure 3:**
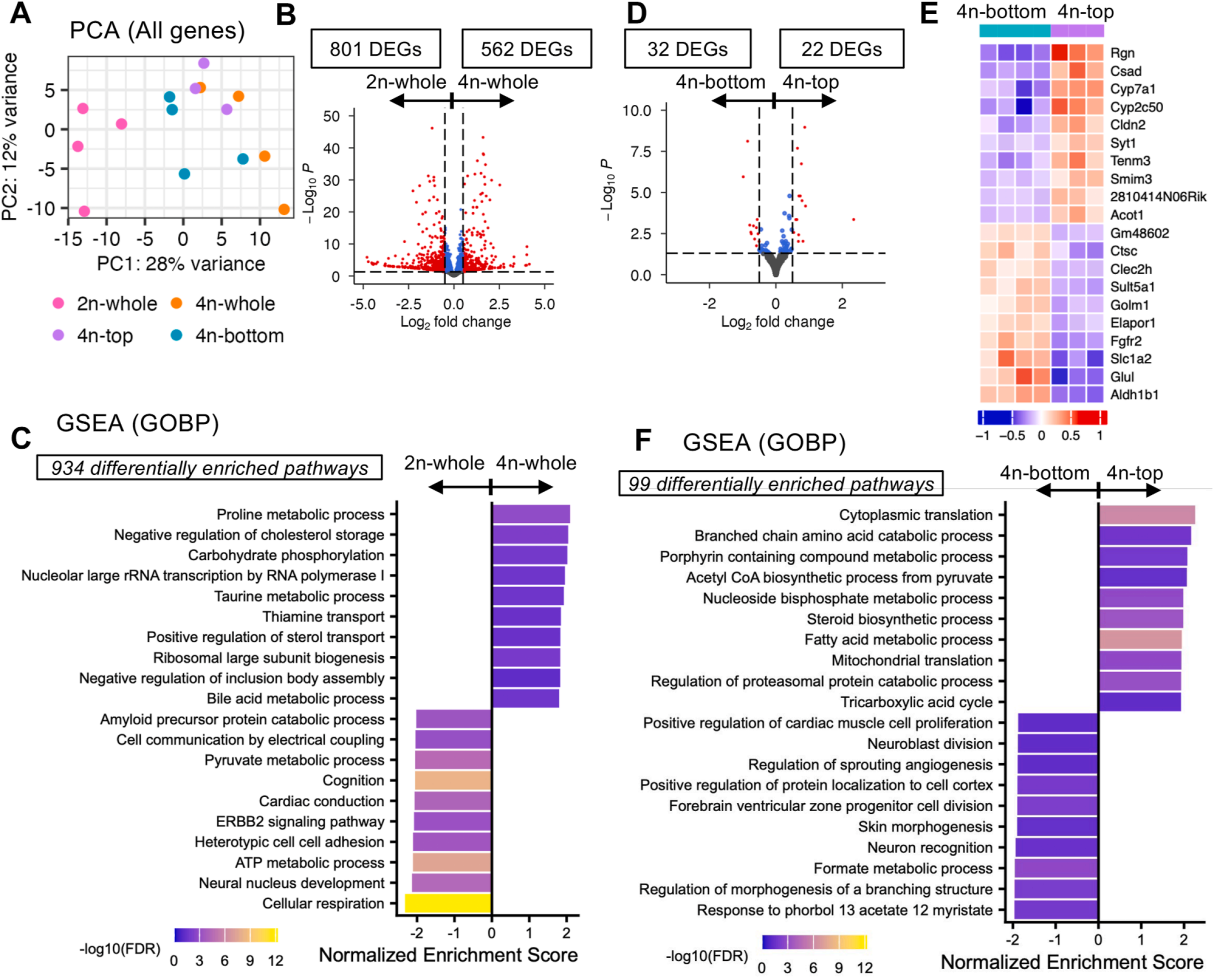
**Transcriptomic analysis of hepatocytes with different ploidy states.***A*, principal component analysis (PCA) using all genes across all samples. *B*, a volcano plot comparing gene expression between 4n-whole and 2n-whole hepatocytes. *C*, representative pathways differentially expressed between 4n-whole and 2n-whole hepatocytes. 10 upregulated and downregulated pathways are shown, respectively. The Gene Ontology Biological Process (GOBP) database was used. FDR, false discovery rate. *D*, a volcano plot comparing 4n-top and 4n-bottom hepatocytes. *E*, a heatmap comparing 4n-top and 4n-bottom hepatocytes. Among the 54 DEGs, the top 10 genes enriched in 4n-top and 4n-bottom are shown, respectively. Genes are colored by z-score normalized expression. *F*, Representative pathways differentially expressed between 4n-top and 4n-bottom hepatocytes. In (*B*) and (*D*), significant genes were defined as p-adj < 0.05 and |log2FC| > 0.5.

We first performed a global principal component analysis (PCA) across all the samples, which revealed a clear separation between the 2n-whole population and the three 4n populations along the first principal component (PC1) (Fig. 3*A*). A binary comparison of the 4n-whole *versus* the 2n-whole population identified 1363 differentially expressed genes (DEGs, p-adj<0.05, |log2FC|>0.5) (Fig. 3*B*, Table S2) and 934 differentially enriched pathways (p-adj<0.05) from the Gene Ontology Biological Process (GOBP) category using Gene Set Enrichment Analysis (GSEA) (differentially expressed pathways shown in Table S3, top enriched pathways shown in Fig. 3*C*) (21, 22, 23, 24, 25). Specifically, the top-ranked pathways enriched for 4n-whole were hepatic function-related, including protein and bile acid metabolism. In contrast, those enriched for the 2n-whole population were hepatic function-unrelated, indicating that 4n hepatocytes exhibited a more mature phenotype, which is consistent with earlier transcriptome analyses (1, 14). These results indicated that our RNA-seq dataset reliably captures the differences between 2n and 4n cells, while representing the overall similarity among the 4n populations.

We next investigated differences between 1x4n (4n-top) and 2x2n (4n-bottom) hepatocytes. Notably, the 4n-bottom population was positioned closer to the 2n-whole population along PC1 than the other 4n populations were (Fig. 3*A*). This observation is consistent with a previous report suggesting that the gene expression profile of 2x2n nuclei is more similar to that of 1x2n nuclei than to that of 1x4n (14). A direct comparison between 4n-top and 4n-bottom populations identified 54 DEGs (Fig. 3*D*, Table S2, representative genes shown in Fig. 3*E*). Moreover, GSEA revealed differential enrichment across multiple pathway categories, including 99 in GOBP (Table S4, top enriched pathways shown in Fig. 3*F*), 14 in KEGG (26, 27, 28), 122 in Reactome (29) and 10 in Hallmark (23) (Fig. S3, Tables S5–S7). Comparing these results with the results between 2n-whole and 4n-whole, it is again suggested that nuclear configurations less influence on the heterogeneity of hepatocytes than cellular ploidy does. Although these differences were substantially smaller than those observed between 2n-whole and 4n-whole hepatocytes, multiple pathways enriched in 4n-top were involved in metabolism, such as *GOBP branched chain amino acid catabolic process* and *GOBP porphyrin-containing compound metabolic process*.

### 1x4n hepatocytes exhibit a stronger metabolic signature than 2x2n hepatocytes, independent of liver zonation

The metabolic activities of hepatocytes vary along the blood flow axis, a phenomenon known as liver zonation (30, 31). Upstream periportal (zone 1) hepatocytes are exposed to higher levels of oxygen and nutrients and are enriched for pathways such as fatty acid β-oxidation, gluconeogenesis, and urea synthesis from amino acids, whereas downstream pericentral (zone 3) hepatocytes preferentially utilize pathways in which oxygen and some nutrients are not required, such as xenobiotic metabolism, glycolysis, and urea synthesis from ammonia (32, 33).

**Figure 4:**
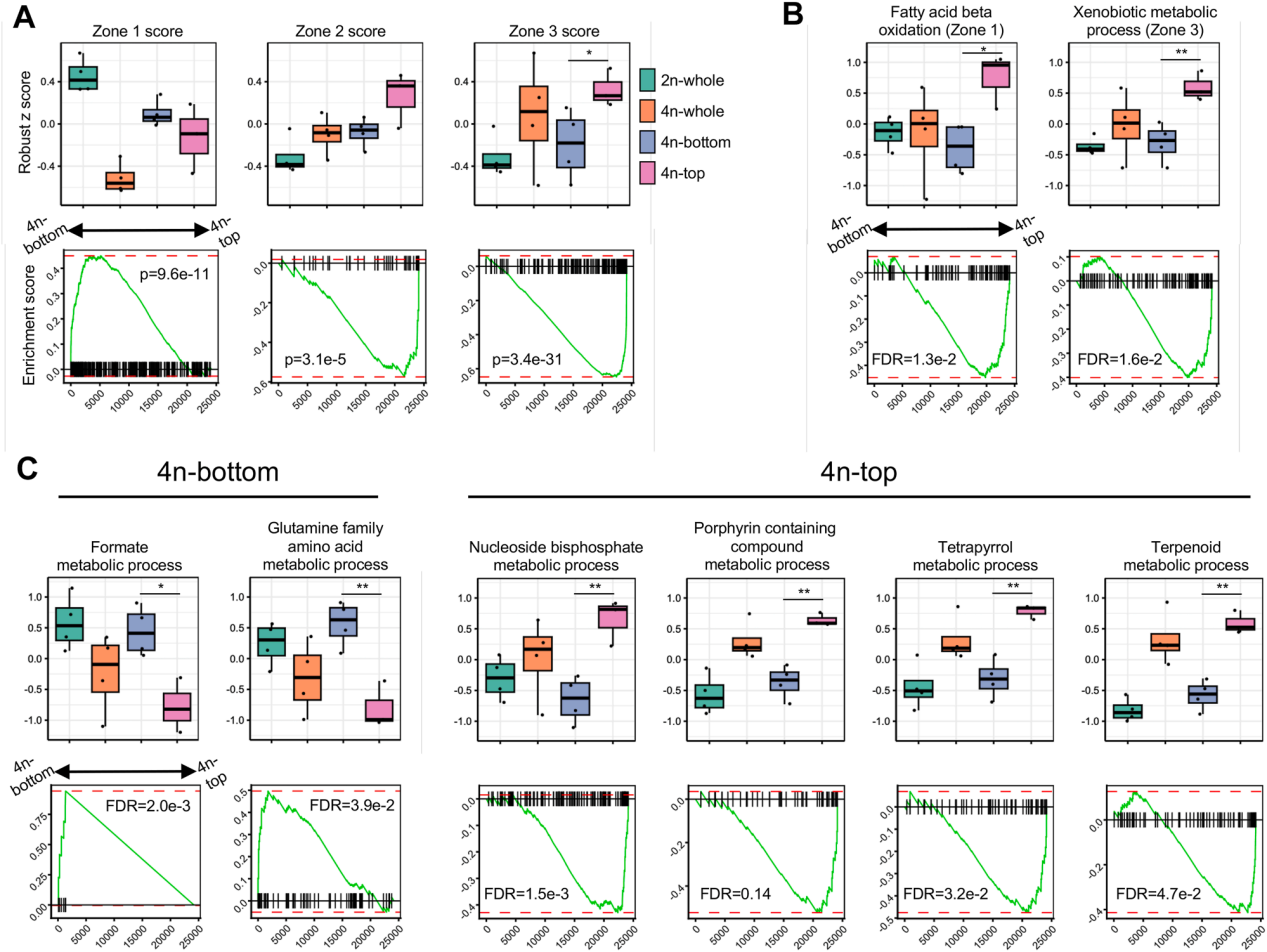
**Relationships between ploidy and zonation.***Upper* and *lower panels* represent robust z-scores and GSEA results of the indicated gene sets, respectively. All populations were used to calculate robust z-scores in the *upper panels* while the *lower panels* compare 4n-bottom and 4n-top populations. *A*, results for manually curated zonation-related gene lists. *B*, Two representative pathways for zone 1 and zone 3, respectively. *C*, results for two pathways enriched in 4n-bottom (left) and representative four pathways enriched in 4n-top (right). FDR, false discovery rate. ∗*p* < 0.05; ∗∗*p* < 0.01.

Given this background, we considered whether these differences were driven by zonation rather than ploidy. To assess the potential influence of zonation, we compiled zone-specific gene lists based on published RNA-seq datasets (31, 34) (Table S8). Robust z-scores and GSEA using these lists revealed that zone 1 genes were enriched in 4n-bottom, while zone 2 and 3 genes were enriched in the 4n-top population (Fig. 4*A*). This result suggests that 2x2n hepatocytes are more prevalent in zone 1, whereas 1x4n hepatocytes are enriched in zone 2 and 3, consistent with previous reports (1, 14, 15).

To further explore the relationship between ploidy and zonation, we examined enrichment of zonation-associated metabolic pathways. Surprisingly, zone 1-related *fatty acid β-oxidation* and zone 3-related *xenobiotic metabolism* were both enriched in 4n-top hepatocytes (Fig. 4*B*). Moreover, the 4n-top population showed enrichment of many other metabolism-related pathways that were not previously linked to zonation (*e.g.*, *nucleoside bisphosphate metabolism*; Fig. 3*F*). In total, more than 20 GOBP pathways representing metabolic processes were found to be statistically significant (Fig. 4*C*, Table S4). In contrast, we found only two metabolic pathways enriched in the 4n-bottom population: *formate metabolism* and *glutamine family amino acid metabolism*. Taken together, these analyses suggested that zonation alone could not fully explain the observed differences between 1x4n and 2x2n hepatocytes. Therefore, it is likely that these two populations possess distinct metabolism-related gene expression programs, with 1x4n hepatocytes exhibiting broader enrichment of metabolic-related pathways.

## Discussion

In previous studies on hepatocyte ploidy, comparisons were primarily made between diploid (2n) and polyploid cells (≥4n) (1, 5, 11, 35, 36). This focus likely reflects the fact that most mammalian somatic cells are diploid, prompting interest in why hepatocytes acquire additional genome copies. However, polyploid hepatocytes can be further subdivided into tetraploid (4n) and octoploid (8n) classes, and additionally into mononucleated and binucleated populations. Notably, mononucleated and binucleated hepatocytes often harbor equivalent total genomic content yet coexist within the liver. This raises the fundamental question of whether there are biological differences between these two populations. However, this question has remained unanswered due to technical limitations in physically separating these cells. This study proposes a FACS-based approach to separate 1x4n and 2x2n hepatocytes. Using this method, we demonstrated that these two populations exhibit overall similarity in their transcriptome compared to diploid hepatocytes, while also revealing previously unknown differences: 1x4n hepatocytes are enriched for a metabolic signature, suggesting higher functionality than 2x2n hepatocytes.

To circumvent the lack of techniques to physically separate 1x4n and 2x2n hepatocytes, recent studies have taken advantage of cutting-edge techniques, including snRNA-seq (14) and scRNA-seq with spatial information (15). In the snRNA-seq study, following FACS sorting of 4n live hepatocytes, their plasma membranes were disrupted, and the nuclei were further sorted to separately yield 1x4n and 2x2n nuclei. By comparing these with the nuclei isolated from 1x2n hepatocytes, the authors proposed that 2x2n nuclei were transcriptionally more similar to 1x2n than to 1x4n nuclei. Our findings are partly consistent with their interpretation in that the 4n-bottom population is more similar to 1x2n hepatocytes than the 4n-top population is (Fig. 3*A*, PC1 axis). However, our results still showed closer similarity of 4n-bottom hepatocytes to 4n-top hepatocytes than to 1x2n hepatocytes, indicating that the nuclear transcriptome does not necessarily predict the corresponding cellular transcriptome, which became clear only through physical sorting of the two populations. Moreover, the snRNA-seq study analyzed a limited number of nuclei (69 1x4n and 66 2x2n), making it difficult to comprehensively assess transcriptional divergence between these populations. Bulk RNA-seq of physically sorted cells, which allows a greater sequencing depth, provides more generalizable conclusions. More recently, scRNA-seq data were obtained from hepatocytes, in which ploidy levels and nuclear configurations were simultaneously determined through nuclear staining with DAPI. The study demonstrated that hepatocytes with higher ploidy or greater nuclear number exhibited increased transcriptional activity. Although this study did not specifically focus on direct comparisons between 1x4n and 2x2n cells, their observation appears to contrast with our finding that the 4n-top population was more enriched for a metabolic signature than the 4n-bottom population. Our transcriptomic analysis using a unique physical sorting approach provides additional insight into the biological differences associated with nuclear configurations of 4n hepatocytes.

Beyond hepatocytes, the present sorting strategy may also be applicable to other multinucleated cell types, such as megakaryocytes, skeletal muscle cells and polyploid cancer cells (37). The biological significance of different nuclear configurations in these cells remains poorly understood. Our approach may provide a useful framework for investigating how nuclear configurations influence cell function across diverse tissue and disease contexts.

Several limitations remain in this study. First, the 4n-top population contained approximately 60% of 1x4n cells, whereas the 4n-bottom population contained approximately 90% of 2x2n cells. This incomplete purity of the 1x4n cells likely attenuated the magnitude of observed transcriptional differences in our RNA-seq results. This study prioritized obtaining a sufficient number of sorted cells to enable reliable bulk RNA-seq as a proof of concept. However, the purity of the 4n-top population can be increased up to 70 to 80% by three rounds of repeated sorting, and simulation results estimated that it could even reach 90% after three to seven rounds. Thus, repeated sorting may further improve the accuracy and sensitivity of detecting transcriptional differences. Second, to improve the purity of 1x4n hepatocytes, additional nuclear markers, such as nuclear envelope protein labeling, could be employed. Theoretically, assuming spherical nuclei of equal volume, the nuclear surface area of 2x2n hepatocytes is 1.26-fold greater than that of 1x4n hepatocytes. This geometric difference may provide an additional parameter for improving discrimination and separation between the two populations. Third, further functional experiments are needed to functionally demonstrate phenotypic differences in cellular metabolic activity between 1x4n and 2x2n hepatocytes. Since physical sorting allows separate isolation of the two populations, harvested cells can be cultured *in vitro*, which may provide a platform for such a direct assessment.

In summary, our novel sorting strategy enabled the selective isolation of 1x4n and 2x2n hepatocytes, allowing us to uncover transcriptomic differences between these two populations. Although more pronounced differences were observed between 4n and 2n cells, 1x4n and 2x2n hepatocytes also exhibited distinct transcriptional signatures, including differential expressions of metabolism-related genes. These findings motivate future work on the roles of different nuclear configurations in cellular function in the liver.

## Experimental procedures

### Mice

C57BL/6JJcl mice were purchased from CLEA Japan. Mice were fed with a standard diet of CE-2 (10 kGy) (CLEA Japan) and kept in a 12-h light-dark cycle. For all experiments, 7 to 15-week-old male mice were used. All mouse experiment procedures used in this study were performed following the University of Tokyo’s guidelines. All mouse procedure protocols used in this study were in accordance with, and with the approval of, the University of Tokyo.

### Hepatocyte isolation

Hepatocyte isolation from whole mouse liver was performed as previously described (38). Briefly, livers were perfused with HBSS (Wako), Liver Perfusion Medium (Gibco), HBSS with 5 mM CaCl2 (Wako) and 500 μg/ml collagenase (Wako) in that order. Following perfusion, livers were mechanically dispersed with tweezers, resuspended in wash medium (DMEM (Wako) with 5% FBS (BioWest)) supplemented with 40 μg/ml DNase (Millipore) and filtered with a 70 μm cell strainer. The cells were centrifuged at 4 °C at 48×*g* for 5 min. Then, the cells were resuspended in complete percoll solution (10.8 ml percoll (Cytiva), 12.0 ml wash medium, and 1.2 ml 10 × HBSS per liver) and centrifuged at 4 °C at 48×*g* for 10 min. After a single wash with wash medium, hepatocytes were used for the downstream experiments.

### Fluorescent staining

Hepatocytes were suspended with DMEM supplemented with 10% FBS and 1x Penicillin-Streptomycin-Amphotericin B Suspension (P.S.A.) (Wako) at a concentration of 2e6/ml. Then, 15 μg/ml Hoechst 33,342 (Wako), 5 μM reserpine (Wako), and 40 μg/ml DNase were added, and hepatocytes were incubated at 37 °C for 30 min while inverted every 10 min. Following centrifugation at 4 °C at 48×*g* for 5 min, hepatocytes were resuspended with flow buffer at a concentration of 1e7/ml. The composition of the flow buffer was HBSS supplemented with 25 mM HEPES (Sigma), 5 mM MgCl_2_ (Wako), 3 mg/ml glucose (Sigma), 1 x P.S.A., 1 x MEM Non-essential Amino Acids Solution (Wako), 1 X GlutaMAX Supplement (Wako), 1 mM sodium pyruvate (Wako), 40 μg/ml DNase, 15 μg/ml Hoechst 33,342, and 5% FBS, pH was adjusted to 7.4 with NaOH (Wako). Then, hepatocytes were filtrated with a 40 μm cell strainer, and 100 nM TO-PRO-3 Iodide (Invitrogen) was added for staining dead cells.

### Flow cytometry

Flow cytometry was performed using SH800S (Sony) with a 130 μm nozzle. Events other than hepatocytes and doublet events were removed by gating on BSC-A *versus* FSC-A and FSC-H *versus* FSC-A, respectively. Dead cells were removed according to TO-PRO-3 fluorescence by gating on FSC-H vs. APC-A. Then, live hepatocytes were plotted by FSC-W *versus* Hoechst 33,342-A or Hoechst 33,342-H *versus* Hoechst 33,342-A. Sorted cells were collected into a 1:1 mixture of DMEM and FBS. After sorting, cells were immediately centrifuged at 4 °C at 48 × *g* for 5 min for 4n and 8n cells, or 800 × *g* for 10 min for 2n cells, respectively. Cells were then resuspended with DMEM supplemented with 10% FBS and 1 x P.S.A. and kept on ice before the downstream experiments.

### Counting of mononucleated and binucleated hepatocytes

A portion of sorted hepatocytes was plated onto a 96-well plate with DMEM supplemented with 10% FBS and 1 x P.S.A. Cells were incubated at 37 °C for a few hours, stained again with 1 μg/ml Hoechst 33,342 and observed by fluorescence microscope (BZ-X810, Keyence). The numbers of mononucleated and binucleated cells were counted, respectively, and the proportions of these cells in sorted samples were calculated.

### *In silico* flow cytometry development

An *in silico* model was developed to simulate fluorescence signals of nuclei stained with Hoechst 33,342 during flow cytometry. Mononucleated and binucleated hepatocytes were modeled as containing either one or two spherical nuclei with homogeneous fluorescence intensity. For binucleated hepatocytes, the angle between the axis connecting the two nuclei and the direction of flow (defined as the z-axis) was randomly assigned according to a uniform distribution. The nuclear volume was assumed to scale proportional with both cellular and nuclear ploidy (1, 20). The length of a nucleus or two nuclei along the z-axis was defined as the width value. Nuclei were divided into 50 segments orthogonal to the z-axis, and the maximum area among the planes was taken as the height value. To incorporate biological and technical variability for each cell, a normal distribution was fitted to the data of each event, with the individual's value as the mean and 0.08 times that value as the standard deviation. The area value for each event was calculated as the product of height and width. The proportion of each ploidy level was based on the experimental results. 20,000 events were measured in total.

The gating boundary separating 4n hepatocytes (4n-whole) into 4n-top and 4n-bottom populations was manually defined. The proportion of mononucleated hepatocytes in the 4n-whole population was assigned as the initial condition for the simulation. The increase in the mononucleated fraction after three sequential rounds of sorting was calculated for both simulated and experimental datasets and compared using *t* test.

To simulate repeated sorting of 4n-top hepatocytes, the proportion of mononucleated cells in the 4n-whole population was fitted to a normal distribution. The initial values of simulation were based on the 95% interval of the fitted distribution. The gating boundary separating 4n hepatocytes (4n-whole) into 4n-top and 4n-bottom populations was manually defined. This procedure was iteratively repeated for up to seven rounds, and the proportion of mononucleated cells was quantified after each round.

### RNA extraction and sequencing

Approximately 80,000 to 300,000 hepatocytes were sorted by flow cytometry. RNA was extracted with FastGene RNA basic kit, including DNase I treatment. The concentration, A260/A280 and A260/A230 of extracted RNA were measured using the NanoDrop One (Thermo). RNA with low purity was cleaned up with the Monarch Spin RNA Cleanup Kit (10 μg) (New England Biolabs). Library preparation and sequencing were performed by Azenta using NovaSeq (Illumina).

### Bioinformatics for RNA-seq

Reads were aligned to the mouse genome (GRCm39) using STAR aligner with default parameters (39). The gene-count matrix was produced by featureCounts (40). Batch effects due to individual differences among mice were adjusted using ComBat-seq (41). Then, to compare gene expression between samples, expression levels were normalized with the median of ratios method using DESeq2, and differential expression was analyzed using the default parameters (42). PCA was performed using all genes. Differentially expressed genes were defined with adjusted *p*-value < 0.05 and |log_2_(fold-change)| > 0.5. The log fold change shrunken was used for the heatmap. Pre-ranked gene set enrichment analysis (GSEA) was performed using fgsea with the DESeq2 test statistic as the ranking parameter (43). Pathways were sourced from the Molecular Signatures Database or as described below (21, 22, 23, 24). Significant pathways were defined using a false discovery rate (FDR) < 0.05. Robust z-scores were calculated by centering expression values to the median and scaling them using the average of the standard deviation and the median absolute deviation.

### Curation of zonation-related gene sets

For zone 1-related and zone 3-related genes, the counts per million matrix from Ben-Moshe *et al.* (PRJNA556572) (34) was used. Eight layers along hepatic zones were merged in pairs to create the expression values for four layers, and the Kruskal–Wallis test was performed for every gene. This yielded 188 zone 1-related and 259 zone 3-related genes by two criteria: p-adj < 0.05 and the median values of the layers exhibit a monotonic increase or decrease. For zone 2-related genes, 46 previously identified genes by Halpern *et al.* were used (31).

## Data availability

Experiment and simulation data will be provided on request. Sequencing data have been deposited to GEO: GSE324432.

## Supporting information

This article contains supporting information (21, 22, 23, 24, 31, 34, 43).

## Conflict of interest

The authors declare that they have no conflicts of interest with the contents of this article.
