# Supplementary material for: A novel sorting method uncovers metabolic heterogeneity between mononucleated and binucleated tetraploid hepatocytes
Source: J Biol Chem. 2026 Jul 13;302(9):113324. doi: 10.1016/j.jbc.2026.113324 (PMC13470166; doi:10.1016/j.jbc.2026.113324)
Supplement: Supplementary file 1 [file mmc1.docx]

**SUPPLEMENTARY INFORMATION**

**Supplementary Figure S1. Additional information of flow cytometry.**

(A) Gating strategy. FSC, forward scatter. SSC, side scatter. (B) Post sorting of each cellular ploidy.

**Supplementary Figure S2. *In silico* flow cytometry.**

The 4n-top population was repeatedly sorted in the simulation.

**Supplementary Figure S3. Additional GSEA results.**

(A) Top enriched pathways by GSEA using KEGG, Hallmark, and Reactome categories. (B-C) GSEA plots comparing 4n-top (left) and 4n-bottom (right). Metabolic pathways enriched in 4n-top (B), and pathways enriched in 4n-bottom (C).

Supplementary tables are put in an excel file "Supplementary_Tables.xlsx". Table legends are below.

**Supplementary Table S1**

The meta data of RNA-seq. The 4n_Top_1 sample was excluded from the downstream analysis due to the poor RNA quality.

**Supplementary Table S2**

The list of DEGs comparing 4-whole and 2n-whole, or 4n-top or 4n-bottom hepatocytes.

**Supplementary Table S3.**

The list of differentially expressed pathways in the 4n-whole relative to 2n-whole population, based on Gene Ontology Biological Process (GOBP). The criterion of the adjusted p value is 0.05.

**Supplementary Table S4**

The list of differentially expressed pathways in the 4n-top relative to 4n-bottom population, based on GOBP. The criterion of the adjusted p value is 0.05.

**Supplementary Table S5**

The list of differentially expressed pathways in the 4n-top relative to 4n-bottom population, based on Kyoto Encyclopedia of Genes and Genomes (KEGG). The criterion of the adjusted p value is 0.05.

**Supplementary Table S6**

The list of differentially expressed pathways in the 4n-top relative to 4n-bottom population, based on Reactome. The criterion of the adjusted p value is 0.05.

**Supplementary Table S7**

The list of differentially expressed pathways in the 4n-top relative to 4n-bottom population, based on Hallmark. The criterion of the adjusted p value is 0.05.

**Supplementary Table S8**

The list of zoanted genes.
